# Association between *Fusobacterium nucleatum* and patient prognosis in metastatic colon cancer

**DOI:** 10.1038/s41598-021-98941-6

**Published:** 2021-10-12

**Authors:** Jii Bum Lee, Kyung-A Kim, Ho Yeon Cho, DooA Kim, Won Kyu Kim, Dongeun Yong, Hyukmin Lee, Sang Sun Yoon, Dai Hoon Han, Yoon Dae Han, Soonmyung Paik, Mi Jang, Han Sang Kim, Joong Bae Ahn

**Affiliations:** 1 Division of Medical Oncology, Department of Internal Medicine, Yonsei Cancer Center, Yonsei University College of Medicine, 50-1 Yonsei-ro, Seodaemun-gu, Seoul, 03722 Korea; 2 Graduate School of Medical Science, Brain Korea 21 Project, Severance Biomedical Science Institute, Yonsei University College of Medicine, Seoul, Korea; 3 Natural Products Research Center, Korea Institute of Science and Technology, Gangnung, Korea; 4 Department of Laboratory Medicine and Research Institute of Bacterial Resistance, Yonsei University College of Medicine, Seoul, Korea; 5 Department of Microbiology and Immunology, Yonsei University College of Medicine, Seoul, Korea; 6 Institute for Immunology and Immunological Diseases, Yonsei University College of Medicine, Seoul, Korea; 7 Department of Surgery, Yonsei University College of Medicine, Seoul, Korea; 8 Department of Pathology, Yonsei University College of Medicine, Seoul, Korea; 9 Department of Pathology, National Health Insurance Service Ilsan Hospital, Goyang, 10444 Korea

**Keywords:** Cancer microenvironment, Colorectal cancer

## Abstract

Recent evidence suggests that *Fusobacterium nucleatum* (*Fn*) is associated with the development and progression of colorectal cancer. We aimed to delineate the clinical implications of *Fn* in metastatic colon cancer. We performed quantitative polymerase chain reaction (qPCR) using DNA samples from synchronous metastatic colon cancer patients with either formalin-fixed paraffin-embedded (FFPE) archival primary site tumor samples or fresh colon tissues. Progression-free survival (PFS)1 and PFS2 were defined as PFS of first- and second-line palliative settings. qPCR for *Fn* was successfully performed using 112 samples (FFPE, n = 61; fresh tissue, n = 51). Forty-one and 68 patients had right-sided and left-sided colon cancer, respectively. Patients with *Fn* enriched right-sided colon cancers had shorter PFS1 (9.7 vs. 11.2 months) than the other subgroups (HR 3.54, 95% confidence interval [CI] 1.05–11.99; *P* = 0.04). *Fn* positive right-sided colon was also associated with shorter PFS2 (3.7 vs. 6.7 months; HR 2.34, 95% CI 0.69–7.91; *P* = 0.04). In the univariate analysis, PFS1 was affected by differentiation and *Fn* positive right-sided colon cancer. The multivariate analysis showed that differentiation (HR 2.68, 95% CI 1.40–5.14, *P* = 0.01) and *Fn* positive right-sided colon (HR 0.40, 95% CI 0.18–0.88, *P* = 0.02) were associated with PFS1. *Fn* enrichment in right sided colon was not associated with overall survival (OS). *Fn* enrichment has significantly worse prognosis in terms of PFS1 and PFS2 in patients with right-sided metastatic colon cancers.

## Introduction

Colorectal cancer (CRC) is the third most commonly diagnosed cancer and the third cause of cancer-related deaths^1^. In patients with stage IV colon cancer, up to 65% of patients experience recurrence after surgical resection^2^. The palliative aim of chemotherapeutic agents such as oxaliplatin and irinotecan in the 5-fluorouracil (5-FU) combination with or without targeted agents has improved median survival^3–5^. Despite systemic treatments with these agents, most patients experience disease progression due to resistance to chemotherapies and targeted agents^6^. The 5-year survival rate in patients with stage IV colon cancer is less than 10%^7^. Therefore, there is a need for a better understanding of the mechanisms of chemoresistance and the development of treatment options in metastatic and recurrent CRC.

Approximately 16% of cancer development is associated with infectious agents^8^. Recently, growing evidence from pre-clinical results showed that the gut microbiome has multiple effects on cancer development, progression, and treatment resistance. Notably, cancer development, and progression of CRC are associated with *Fusobacterium nucleatum* (*Fn*), a gram-negative anaerobe found to be enriched in both primary colon cancer and distant metastatic sites^9–12^. Clinically, *Fn* infection is associated with decreased T-cell infiltration, *BRAF* mutation, tumor-infiltrating macrophages, and microsatellite instability-high (MSI-H)^13–16^. Moreover, an increased amount of *Fn* also correlates with poor overall survival (OS) in advanced stages and is more prevalent in the right-sided colon^17,18^.

Several mechanisms of *Fn*’s role in chemoresistance in CRC have been suggested in pre-clinical models^19^. *Fn* promotes carcinogenesis through surface adhesion virulence factors such as FadA and Fap2, which inhibit the activity of immune cells^20,21^. Furthermore, *Fn* harbors chemoresistance by modulating the autophagy pathway by targeting innate immune signaling^11^. Despite these findings, the role of *Fn* on chemoresistance remains elusive in the clinical setting.

In this study, we aimed to delineate the clinical implications of *Fn* in patients with synchronous metastatic colon cancer. To this end, we used surgical specimens to perform quantitative polymerase chain reaction (qPCR), detect *Fn*, and analyze the prognostic role of *Fn*. We determined whether *Fn* enrichment by primary tumor location (left-sided vs. right-sided) was correlated with patient prognosis.

## Results

### Baseline characteristics according to *Fn* enrichment

**Figure 1:**
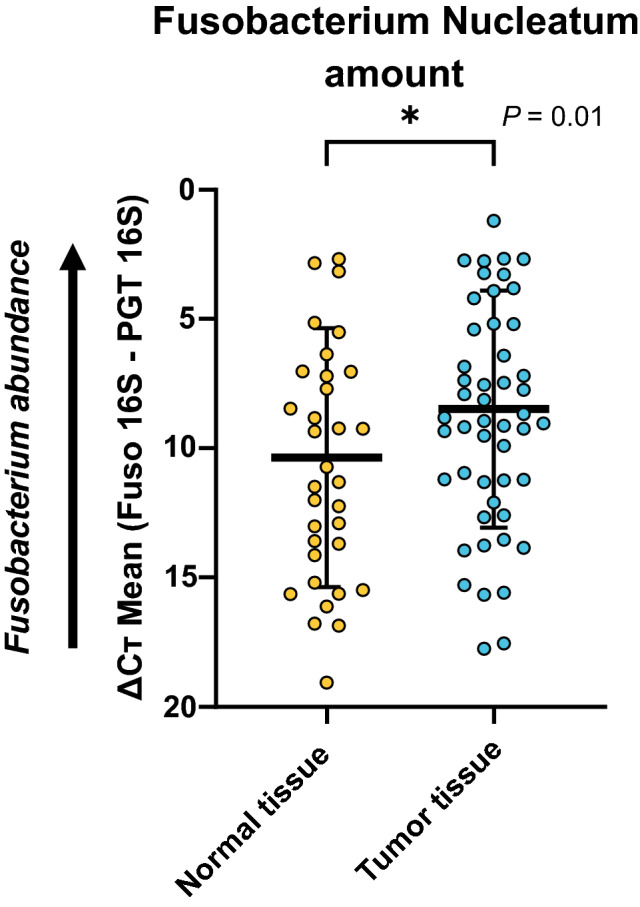
Relative amount of *Fusobacterium nucleatum* (*Fn*) in the tumor (n = 34, ΔCT mean: 8.47) tissue and adjacent normal (n = 51, ΔCT mean: 10.36) tissues. Asterisk indicates significant differences between the ΔCT mean of the tumor and adjacent normal tissues (*P < 0.05).

Among the patients with synchronous metastatic colon cancer, qPCR was successfully performed using 112 patient colon tumor samples, including 61 FFPE and 51 fresh tissue samples. We also analyzed fresh tumor specimens with adjacent normal tissue (n = 34), as shown in Fig. 1. Compared to adjacent normal tissues, tumor tissues had four times higher level of *Fn* (*P* = 0.01).

Baseline patient characteristics with both *Fn* enriched (n = 44) and *Fn* negative (n = 68) metastatic colon cancer are shown in Table 1. Patients with right-sided colon cancer accounted for 37% (n = 41), and majority were diagnosed with left-sided colon cancer (n = 68, 61%). Most of the patients were classified as having adenocarcinoma (n = 108, 96%). The moderately differentiated (MD, n = 74, 66%) grade was the most common pathologic differentiation, followed by the well- (WD, n = 25, 22%) and poorly (PD, n = 13, 12%) differentiated grades. The *KRAS, NRAS,* and *BRAF* mutations were not significantly different between the two subgroups, with *P*-values of 0.77 and 0.58, respectively. There was also no difference in microsatellite status between the two groups (*P* = 0.13), although only 50 patients (45%) had results for evaluation. Most patients had three or more metastatic sites (n = 98, 87%) prior to palliative systemic chemotherapy. Fifteen patients (13%) did not receive palliative chemotherapy. Among the patients treated in first-line palliative setting, most patients (n = 66, 59%) were administered FOLFIRI (bolus and infused fluorouracil with irinotecan), followed by FOLFOX (bolus and infused fluorouracil with oxaliplatin; n = 24, 21%;), and others (n = 7, 7%) which included investigational agents (n = 2), capecitabine (n = 2), CAPOX (capecitabine combined with oxaliplatin; n = 1), and 5-FU infusion with leucovorin (n = 1). Most of the patients were not treated with targeted agents (n = 74, 66%). Bevacizumab was administered to 32 patients (29%) and cetuximab to 6 patients (5%). Overall, there were no identifiable factors that differed between the two groups. Second and third-line chemotherapy was administered to 61 (54%) and 31 patients (28%), respectively. The median follow-up duration after the initial diagnosis was 2.2 years. At the time of analysis, 71 patients (63%) were deceased.

**Table 1:** Baseline characteristics of patients.

	*Fusobacterium nucleatum*			
	Total	Negative	Positive	P value
Number of patients	112	68	44	
**Age (years)**				
< 65	49 (44%)	32 (65%)	17 (35%)	0.44
≥ 65	63 (56%)	36 (57%)	27 (43%)	
**Sex**				
Male	66 (59%)	39 (59%)	27 (41%)	0.7
Female	46 (41%)	29 (63%)	17 (37%)	
**Location**				
Right	41 (37%)	30 (73%)	11 (27%)	0.09
Left	68 (61%)	37 (54%)	31 (46%)	
Both right and left	3 (2%)	1 (33%)	2 (67%)	
**Histology**				
Adenocarcinoma	108 (96%)	65 (60%)	43 (40%)	0.99
Mucinous	4 (4%)	3 (75%)	1 (25%)	
**Differentiation**				
WD	25 (22%)	16 (64%)	9 (36%)	0.77
MD	74 (66%)	43 (58%)	31 (42%)	
PD	13 (12%)	9 (69%)	4 (31%)	
***KRAS/NRAS***				
Mutated	35 (31%)	21 (60%)	14 (40%)	0.77
Wild type	61 (54%)	36 (59%)	25 (41%)	
NA	16 (15%)	11 (69%)	5 (31%)	
**BRAF**				
Mutated	5 (4%)	2 (40%)	3 (60%)	0.58
Wild	98 (88%)	61 (62%)	37 (38%)	
NA	9 (8%)	5 (56%)	4 (44%)	
**Microsatellite status**				
MSI-H	1 (1%)	0 (0%)	1 (100%)	0.13
MSS	49 (44%)	26 (53%)	23 (47%)	
NA	62 (55%)	42 (68%)	20 (32%)	
**Number of metastatic sites**				
< 2	14 (13%)	7 (50%)	7 (50%)	0.4
≥ 3	98 (87%)	61 (62%)	37 (38%)	
**First-line palliative chemotherapy**				
FOLFOX	24 (21%)	13 (54%)	11 (46%)	0.80
FOLFIRI	66 (59%)	40 (61%)	26 (39%)	
Others	7 (7%)	5 (71%)	2 (39%)	
None	15 (13%)	10 (67%)	5 (33%)	
**First-line targeted agents**				
Bevacizumab	32 (29%)	19 (59%)	13 (41%)	0.83
Cetuximab	6 (5%)	3 (50%)	3 (50%)	
None	74 (66%)	46 (62%)	28 (38%)	
**Subsequent chemotherapy**				
Second line	61 (54%)	24 (39%)	37 (61%)	0.506
Third line	31 (28%)	10 (32%)	21 (68%)	

*WD* well differentiated, *MD* moderately differentiated, *PD* poorly differentiated, *MSI-H* microsatellite instability-high, *MSS* microsatellite stable, *FOLFOX* bolus and infused fluorouracil with oxaliplatin, *FOLFIRI* bolus and infused fluorouracil with irinotecan.

### *Fn* and patient prognosis in metastatic colon cancer

We examined whether progression-free survival (PFS) and overall survival (OS) differed between subgroups to test our hypothesis whether enrichment in *Fn* by tumor location (right- and left-sidedness) had prognostic implications in metastatic and recurrent colon cancer. PFS1, PFS2, and PFS3 were defined as PFS of first-, second-, and third-line palliative treatment settings. The subgroups were categorized according to *Fn* enrichment and primary tumor location (right or left-sidedness). Synchronous right- and left-sided colon cancers (n = 3, 2%) were excluded from the analysis.

**Figure 2:**
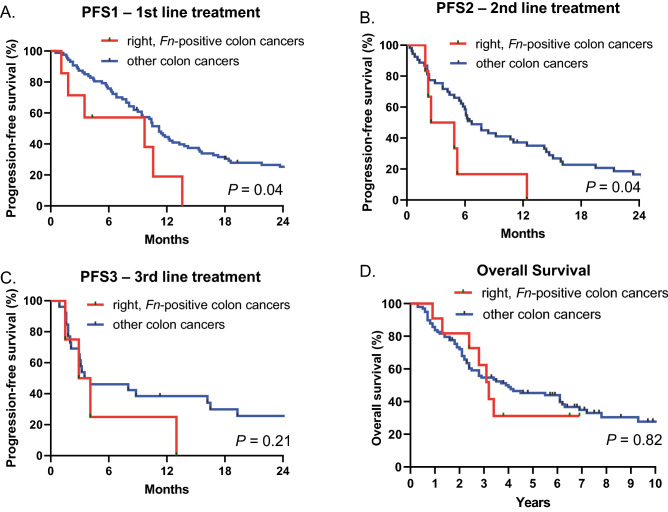
Kaplan–Meier curves of progression-free survival (PFS) in the (**A**) first (PFS1)-, (**B**) second (PFS2)-, (**C**) third (PFS3)-line palliative treatment, and (**D**) overall survival (OS) for *Fn* enriched, right-sided colon versus others. Others include (1) *Fn* enriched, left-sided colon cancer, (2) *Fn* negative, right-sided colon cancer, and (3) *Fn* negative, left-sided colon cancer.

Patients with *Fn* enriched right-sided colon cancers (n = 11) had shorter median PFS1(mPFS1) than other subgroups did (9.7 vs. 11.2 months, respectively, HR 3.54, 95% CI 1.05–11.99; *P* = 0.04) (Fig. 2A). Other subgroups (n = 98) included *Fn* negative, right-sided colon cancer (n = 30), *Fn* enriched, left-sided colon cancer (n = 31), and *Fn* negative, left-sided colon cancer (n = 37). Subsequently, 59 and 30 patients received second and third line palliative chemotherapy, respectively. Patients with *Fn* enriched right-sided colon cancers (n = 6) also had shorter median PFS2 (mPFS2) compared to other subgroups (n = 53) (3.7 vs. 6.7 months, respectively, HR 2.34, 95% CI 0.69–7.91; *P* = 0.04) (Fig. 2B). There was no statistical significance in median PFS3 (mPFS3) between *Fn* enriched right-sided colon cancers (n = 4) and other subgroups (n = 26) (Fig. 2C). Although there was a trend for shorter median OS (mOS) of 3.2 years in *Fn* enriched right-sided colon cancers compared to other subgroups with a mOS of 4 years, there was no statistical significance between the two groups (hazard ratio [HR] 1.10, 95% confidence interval [CI] 0.48–2.51; *P* = 0.82) (Fig. 2D).

We then compared mPFS1, mPFS2, mPFS3, and mOS among the four groups (Supplementary Fig. S1A). *Fn*-enriched right-sided colon cancers had the shortest mPFS1 of 9.7 months, whereas *Fn-*enriched left-sided colon had the longest mPFS1 of 14.5 months (*P* = 0.02). Although the difference in mPFS1 was not statistically significant (*P* = 0.20), the mPFS1 in patients with *Fn* negative right-sided colon cancer (10.4 months) was comparable to that in patients with left-sided colon cancer without *Fn* enrichment (10.2 months). Similar trends were seen in mPFS2 (*P* = 0.19) and mPFS3 (*P* = 0.48), with *Fn*-enriched right-sided colon cancers with the shortest PFS, but the results were not statistically significant (Supplementary Fig. S1B,C).

Patients were also categorized according to first-line palliative treatment received and were grouped as (1) *Fn* enriched, FOLFIRI-treated colon cancer, (2) *Fn* negative, FOLFIRI-treated colon cancer, (3) *Fn* enriched, FOLFOX-treated colon cancer, and (4) *Fn* negative, FOLFOX-treated colon cancer (Supplementary Fig. S2A,B). Although *Fn* negative, FOLFOX treated colon cancer had the shortest PFS and OS, there was no statistical difference of PFS1 (*P* = 0.34) and OS (*P* = 0.23) among the subgroups. Patients with *Fn* enriched right-sided colon cancer had the shortest OS of 3.2 years, followed by patients with *Fn* enriched left-sided colon cancer (3.7 years), *Fn* negative right-sided colon cancer (3.9 years), and *Fn* negative left-sided colon cancer (4.1 years). Overall, the mOS was similar across all subgroups (*P* = 0.99) (Supplementary Fig. S1D).

### Univariate and multivariate analyses of factors affecting survival

We performed a univariate analysis of the factors affecting PFS1 and OS. The univariate analysis of PFS1 showed that tumor differentiation (HR 0.89, 95% CI 9.46–12.94, *P* = 0.02) and *Fn* positive right-sided colon cancer (HR 4.80, 95% CI 0.32–19.15, *P* = 0.02) were associated with worse PFS (Table 2). The multivariate analysis further showed that both differentiation (HR 2.68, 95% CI 1.40–5.14, *P* = 0.01) and *Fn* positive right-sided colon cancer (HR 0.40, 95% CI 0.18–0.88, *P* = 0.02) were significant determinants of PFS1. In both univariate and multivariate analyses, *Fn* enriched right-sided colon cancer was not an independent risk factor of OS (Supplementary Table 1). Tumor differentiation was an independent factor in both univariate (HR 1.31, 95% CI 1.57–6.73) and multivariate analyses (HR 4.00, 95% CI 0.18–0.88, *P* = 0.02).

**Table 2:** Univariate and multivariate analyses of progression-free survival (PFS)1 in the first-line treatment.

Variables	Category	Univariate			Multivariate		
		HR	95% CI	P	HR	95% CI	P
Age	< 65 vs. ≥ 65 years	0.82	9.59–12.81	0.63			
Differentiation	WD, MD vs. PD	0.89	9.46–12.94	0.02	2.68	1.40–5.14	0.01
Number of metastatic sites	< 2 vs. ≥ 3	4.89	0–18.96	0.86			
*Fn* positive, right-sided colon vs. others		4.80	0.32–19.15	0.02	2.51	1.14–5.56	0.02

*WD* well differentiated; *MD* moderately differentiated; *PD* poorly differentiated; *PFS1* progression-free survival1; *Fn*, *Fusobacterium nucleatum*; *HR* hazard ratio, *CI* confidence interval.

### Validation in The Cancer Genome Atlas (TCGA) metastatic colon cancer

**Figure 3:**
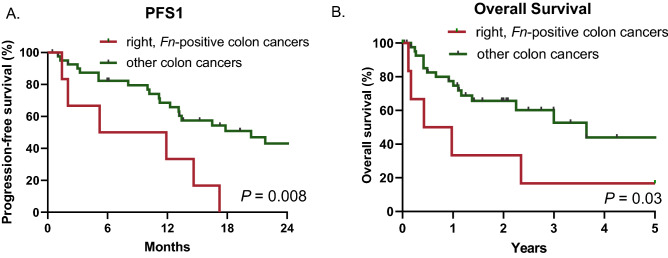
Kaplan–Meier curves of (**A**) progression-free survival (PFS) and (**B**) overall survival (OS) for *Fn* enriched, right-sided colon versus others in the validation cohort using The Cancer Genome Atlas (TCGA) colon cancer data. Others include (1) *Fn* enriched, left-sided colon cancer, (2) *Fn* negative, right-sided colon cancer, and (3) *Fn* negative, left-sided colon cancer.

Next, we applied our findings to a large cancer genome cohort as independent validation^22^. In the TCGA colon cancer cohort, a total of 53 patients was stage IV metastatic colon cancer. Consistent with our finding, patients with *Fn* enriched right-sided colon cancers (n = 7) had shorter median PFS1 (mPFS1) than other subgroups did (8.5 vs. 20.4 months; HR 6.3, 95% CI 1.59–25.08; log-rank test, *P* = 0.008) (Fig. 3A and Supplementary Fig. S3A). Furthermore, patients who harbor *Fn* enriched right-sided colon cancers (n = 7) were significantly associated with shorter OS (8.4 months vs. 3.6 years; HR 4.6, 95% CI 1.10–19.19; log-rank test, *P* = 0.036) (Fig. 3B and Supplementary Fig. S3B).

## Discussion

The gut microbiome affects carcinogenesis, progression, and response to treatment in CRC^19^. Among diverse microbiomes, *Fn* is one of the most commonly found in tissue samples of colon cancers and contributes to chemoresistance^11,23^. Similarly, our study showed that *Fn* was enriched four-fold higher in colon tissues than in normal adjacent tissues. *Fn* enriched right-sided colon cancer was associated with a poor prognosis in terms of mPFS. In contrast, *Fn* negative right-sided colon cancer had comparable mPFS and mOS with left-sided colon cancer. In addition to the tumor right-sidedness, *Fn* positivity contributed to the lack of response to systemic chemotherapy in palliative settings. Collectively, these findings suggest that *Fn* contributes to chemoresistance and may be a potential predictive or prognostic biomarker in metastatic and recurrent rectal cancer.

Although the previous report suggests that the amount of tissue *Fn* was associated with poor cancer-specific mortality^17,24^, the data on cancer treatment were limited, and the role of *Fn* on chemoresistance was not addressed in clinical samples. Therefore, our study tried to add a missing link between chemoresistance of *Fn* in preclinical studies^11,23^ and the clinical validation. Considering that the development of chemoresistance is a critical issue in metastatic colon cancer, we focused on synchronous metastatic colon cancer patients. We showed shorter PFS1 and PFS2 in metastatic patients than other reports with heterogeneous colorectal cancer patients with all tumor stages^17,24^.

Several studies have shown that primary tumor location affected prognosis in patients with right-sided colon cancer having worse prognosis in recurrent and metastatic settings^25,26^. *Fn* has been found in abundance in proximal locations such as cecum, ascending, and transverse colon compared to distal locations, including sigmoid colon and rectum^18^. One potential mechanism underlying chemoresistance to 5-FU and oxaliplatin is the ability of *Fn* to target innate immune signaling pathways such as Toll-like receptor 4 (TLR4) and activating Myeloid differentiation primary response 88 (MyD88) in cancer cells to activate autophagy pathway^11^. The abundance and negative prognostic implications of *Fn* in right-sided colon cancer warrant treatment strategies specifically targeting *Fn*. Treatment with metronidazole for *Fn* enriched patient-derived xenograft showed a decrease in both the amount of *Fn* and tumor growth^12^. However, metronidazole does not target only *Fn* but also normal anaerobes in the intestines, which may disrupt the composition of normal flora^27^. Thus, *Fn*-specific treatment with antimicrobial agents is limited in practicality.

The presence of *Fn* is associated with microsatellite instability (MSI-high) and shorter survival^13,14,17,28^. Two virulence factors, Fap2 and FadA, have been identified as potential immune modulators^20,21^. Fap2 protein binds to T cell immunoreceptor with Ig and ITIM domain and suppresses natural killer cell cytotoxicity^21^. FadA binds to vascular endothelial cadherin (CDH5) and E-cadherin^20^. The binding to CDH5 receptors activates the inflammatory genes of NF-κB and cytokines, and the binding to E-cadherin expressed on colon cells activates *Wnt* genes and oncogenes, thereby promoting an immune-suppressive environment.

Until recently, the current standard of palliative treatment in colon cancer was limited to cytotoxic agents with targeted agents such as cetuximab and bevacizumab^5^. Recently, an anti-PD-1 agent pembrolizumab showed superior efficacy in MSI-H/mismatch repair deficient metastatic CRC based on the pivotal Keynote-177 trial^29^. The study demonstrated the doubling of PFS in response to pembrolizumab compared to standard chemotherapy with or without bevacizumab or cetuximab^29^. Although MSI-H incidence in metastatic CRC setting is less than 5%, pembrolizumab is a promising new standard of treatment option for this subset of patients^30^. Whether treatment with immune checkpoint inhibitors such as pembrolizumab is more effective in *Fn* enriched colon cancer harboring MSI-H and whether changes in enrichment status of *Fn* are evident after immunotherapy are interesting questions to be studied in the future. In our study, 55% of patients did not have MSI status because we retrospectively reviewed and included surgical specimens that date back to the time when MSI was not performed routinely. Only one patient with MSI-H colon cancer was included and treated with cytotoxic chemotherapy.

Limitations to this study include small sample size and its retrospective nature. Although we collected 112 tissue samples, further analysis of primary tumor location and *Fn* positivity resulted in fewer patients and uneven distribution in subgroups. Considering shorter PFS in response to palliative chemotherapy and poor prognosis of *Fn* positivity in both our cohort and independent TCGA metastatic colon cancer data, a larger number of patients in prospective settings warrants further validation and expansion of our findings.

In conclusion, *Fn* enriched right-sided metastatic, and recurrent colon cancer was significantly associated with worse PFS, indicating that *Fn* enriched right-sided colon responded less to palliative cytotoxic chemotherapy. Further analysis, including more extensive patient sampling and prospective cohort, are needed to validate the proposed role of *Fn* in chemoresistance.

## Methods

### Study population

From January 2009 to July 2019, data of patients with metastatic and recurrent colon cancer in Yonsei Cancer Center, Korea, were collected retrospectively. A total of 112 patients had surgical colon specimens available either as Formalin-Fixed Paraffin-Embedded (FFPE) or fresh tissue for analysis of *Fn* using qPCR.

Clinicopathological variables including age, sex, primary tumor location, histology, sites of metastasis, mutations such as *KRAS, NRAS, BRAF*, and microsatellite status were collected. The staging was determined using the 8th edition of the American Joint Committee on Cancer guideline of the primary tumor, node, and metastasis classification^31^. Right-sided colon cancers were defined as tumors occurring from cecum to transverse colon, and left-sided colon cancers as tumors arising from splenic flexure to sigmoid colon^26^.

Patients were treated with first-line palliative chemotherapeutic agents such as FOLFOX (5-fluorouracil, leucovorin, and oxaliplatin), FOLFIRI (5-fluorouracil, leucovorin, and irinotecan) with or without targeted agents such as bevacizumab or cetuximab, CAPEOX (capecitabine and oxaliplatin), and capecitabine. These regimens have been described in detail in another study^32^. Response evaluation was performed using computed tomography scanning every two months, every four cycles for FOLFOX and FOLFIRI, and every three cycles for capecitabine.

All authors followed Good Clinical Practice, and the study was conducted according to the principles of the Declaration of Helsinki. All enrolled patients provided written informed consent. The protocol was approved by the Institutional Review of Severance Hospital (IRB 4-2014-0239).

### DNA isolation and quantitative PCR analysis of *Fn*

Genomic DNA was isolated from clinical samples using the QIAamp DNA Mini Kit (Qiagen, Crawley, UK). Briefly, the samples were suspended with protease K in ATL buffer and incubated at 55 °C for 2 h. Both AL buffer and absolute ethanol were added to the samples before applying the QIAamp spin column. Each sample was centrifuged and washed according to the manufacturer’s protocol. DNA was eluted from the column with 50 μL of the supplied AE buffer. The quality and quantity of the isolated DNA were determined using a NanoDrop spectrophotometer (ND-1000; Thermo Scientific, MA, USA).

To detect the *Fn* sequence, specific primers and probes were designed to recognize the 16S ribosomal RNA gene. Amounts of *Fn* DNA were determined using quantitative real-time PCR using the TaqMan assay system. Each reaction was carried out in the final volume of 20 μL reaction containing 1 × TaqMan Universal PCR Master Mix (Applied Biosystems, CA, USA), 300 nM of each primer, 200 nM TaqMan probe, and 30 ng of genomic DNA in a 96-well optical PCR plate. Amplification, detection, and data analysis were performed using the StepOnePlus Real-Time PCR Systems (Applied Biosystems, CA, USA) as follows: 10 min pre-incubation at 95 °C, amplification cycles of 95 °C for 15 s and 60 °C for 1 min were repeated 50 times. The cycle threshold (Ct) values for *Fn* were normalized to the amount of gDNA in each reaction using prostaglandin transporter (PGT) as a human reference gene.

The primer and probe sequences for the TaqMan assay system were as follows: *Fn* forward primer, 5′-GGATTTATTGGGCGTAAAGC-3′; *Fn* reverse primer, 5′-GGCATTCCTACAAATATCTACGAA-3′; *Fn* FAM probe, 5′-CTCTACACTTGTAGTTCCG-3′; PGT forward primer, 5′-ATCCCCAAAGCACCTGGTTT-3′; PGT reverse primer, 5′-AGAGGCCAAGATAGTCCTGGTAA-3′; PGT FAM probe, 5′-CCATCCATGTCCTCATCTC-3′.

### Statistical analysis

For all statistical analysis, differences were considered to be statistically significant at *P* < 0.05. Baseline characteristics were compared using the Kruskal–Wallis test or Mann–Whitney U test. Progression-free survival (PFS) was defined as the time from the start of palliative chemotherapy to the date of progression, last follow-up, or death. The time points for PFS1, PFS2, and PFS3 were defined as the start of first-, second-, and third-line palliative chemotherapy, respectively. OS was calculated from the date of initial diagnosis to the date of last follow-up or death. The Kaplan–Meier method was used to estimate median PFS and OS, and the Cox proportional hazard model was used for multivariate analysis. All analyses were conducted using SPSS statistical software ver. 25 (IBM, Chicago, IL, USA) and GraphPad Prism 8 (GraphPad Software, Inc., San Diego, CA).

## Supplementary Information

Supplementary Table 1.

Supplementary Figures.

## Data Availability

The datasets used or analyzed from this trial may be available upon reasonable request to the corresponding author.
