# Supplementary material for: Association between Fusobacterium nucleatum and patient prognosis in metastatic colon cancer
Source: Sci Rep. 2021 Oct 12;11:20263. doi: 10.1038/s41598-021-98941-6 (PMC8511250; doi:10.1038/s41598-021-98941-6)
Supplement: Supplementary file 1 — Supplementary Table 1. [file 41598_2021_98941_MOESM1_ESM.docx]

**Supplementary Table 1.** Univariate and Multivariate Analyses of OS

| **Variables** | **Category** | **Univariate** | | | **Multivariate** | | |
| --- | --- | --- | --- | --- | --- | --- | --- |
|  |  | **HR** | **95% CI** | **p** | **HR** | **95% CI** | **p** |
| Age | <65 vs. >65 years | 0.67 | 2.55–5.19 | 0.74 |  |  |  |
| Differentiation | WD, MD vs. PD | 1.31 | 1.57–6.73 | **0.01** | 4.00 | 2.07–7.75 | **0.01** |
| Number of metastatic sites | <2 vs. >3 | 2.15 | 0–8.36 | 0.96 |  |  |  |
| *Fn* positive, right-sided colon vs*.* others |  | 0.28 | 2.66–3.78 | 0.80 |  |  |  |

**Abbreviations:** WD, well differentiated; MD, moderately differentiated; PD, poorly differentiated; OS, overall survival; *Fn, Fusobacterium nucleatum*; CI, confidence interval
