# Supplementary material for: Association between Fusobacterium nucleatum and patient prognosis in metastatic colon cancer
Source: Sci Rep. 2021 Oct 12;11:20263. doi: 10.1038/s41598-021-98941-6 (PMC8511250; doi:10.1038/s41598-021-98941-6)
Supplement: Supplementary file 2 — Supplementary Figures. [file 41598_2021_98941_MOESM2_ESM.pptx]

## Slide 1
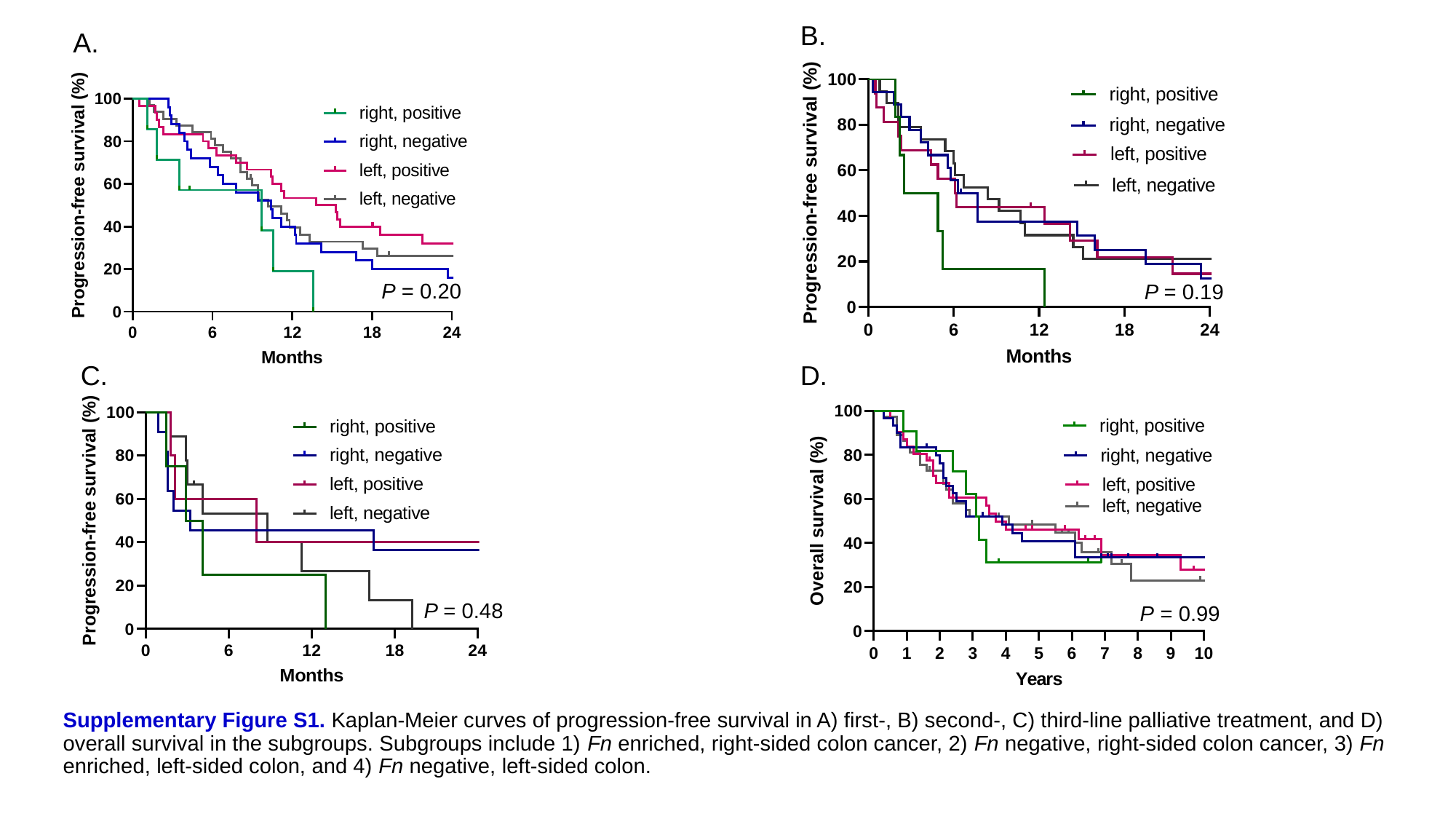

B.
A.
P = 0.20
P = 0.19
C.
D.
P = 0.48
P = 0.99
# Supplementary Figure S1. Kaplan-Meier curves of progression-free survival in A) first-, B) second-, C) third-line palliative treatment, and D) overall survival in the subgroups. Subgroups include 1) Fn enriched, right-sided colon cancer, 2) Fn negative, right-sided colon cancer, 3) Fn enriched, left-sided colon, and 4) Fn negative, left-sided colon.

## Slide 2
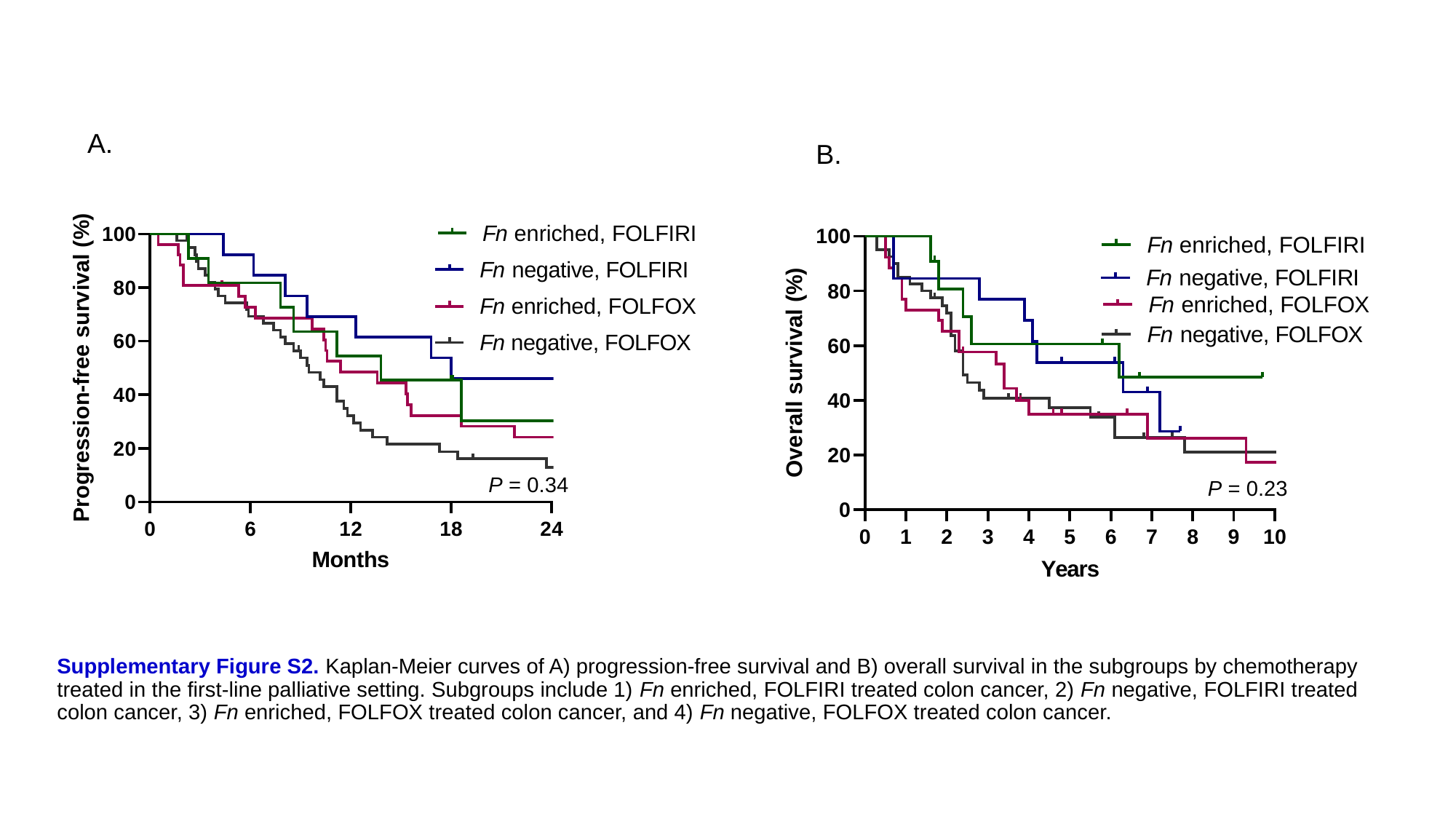

A.
B.
P = 0.34
P = 0.23
Supplementary Figure S2. Kaplan-Meier curves of A) progression-free survival and B) overall survival in the subgroups by chemotherapy treated in the first-line palliative setting. Subgroups include 1) Fn enriched, FOLFIRI treated colon cancer, 2) Fn negative, FOLFIRI treated colon cancer, 3) Fn enriched, FOLFOX treated colon cancer, and 4) Fn negative, FOLFOX treated colon cancer.

## Slide 3
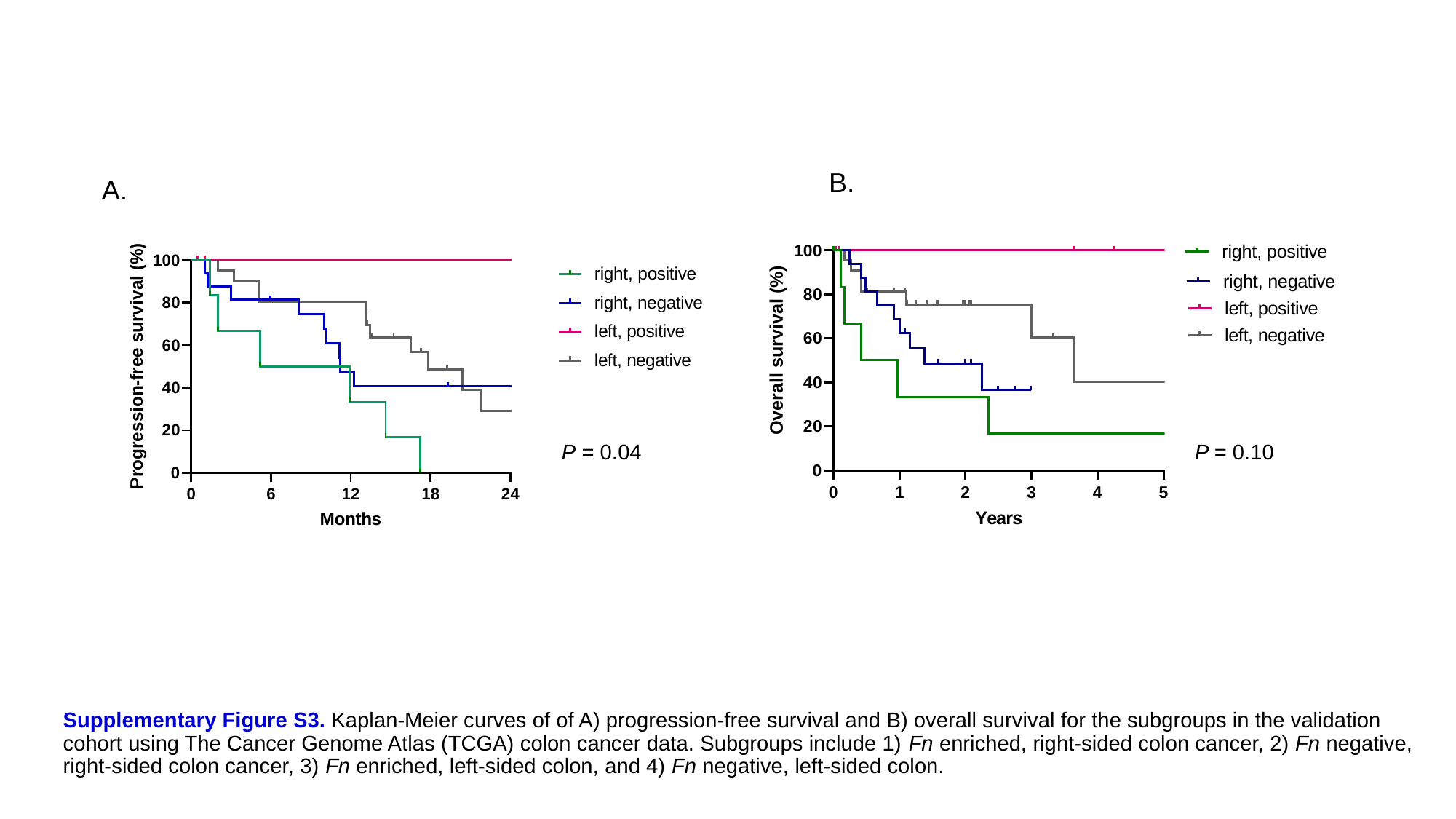

B.
A.
P = 0.04
P = 0.10
# Supplementary Figure S3. Kaplan-Meier curves of of A) progression-free survival and B) overall survival for the subgroups in the validation cohort using The Cancer Genome Atlas (TCGA) colon cancer data. Subgroups include 1) Fn enriched, right-sided colon cancer, 2) Fn negative, right-sided colon cancer, 3) Fn enriched, left-sided colon, and 4) Fn negative, left-sided colon.
